# Questionnaire survey of detrimental fur animal epidemic necrotic pyoderma in Finland

**DOI:** 10.1186/s13028-017-0322-z

**Published:** 2017-08-03

**Authors:** Heli Nordgren, Katariina Vapalahti, Olli Vapalahti, Antti Sukura, Anna-Maija Virtala

**Affiliations:** 1 Department of Veterinary Biosciences, Faculty of Veterinary Medicine, University of Helsinki, Helsinki, Finland; 2 Department of Virology and Immunology, HUSLAB, Hospital district of Helsinki and Uusimaa, Helsinki, Finland; 3 Department of Virology, Faculty of Medicine, University of Helsinki, Helsinki, Finland

**Keywords:** *Arcanobacterium phocae*, Fur animals, Fur animal epidemic necrotic pyoderma FENP, *Neovison vison*, *Nyctereutes procyonoides*, *Vulpes lagopus*

## Abstract

**Background:**

In 2007, a previously unrecorded disease, fur animal epidemic necrotic pyoderma (FENP), was detected in farmed mink (*Neovision vision*), foxes (*Vulpes lagopus*) and Finnraccoons (*Nyctereutes procyonoides*) in Finland. Symptoms included severe pyoderma with increased mortality, causing both animal welfare problems and economic losses. In 2011, an epidemiologic questionnaire was mailed to all members of the Finnish Fur Breeders’ Association to assess the occurrence of FENP from 2009 through the first 6 months of 2011. The aim was to describe the geographical distribution and detailed clinical signs of FENP, as well as sources of infection and potential risk factors for the disease.

**Results:**

A total of 239 farmers (25%) returned the questionnaire. Clinical signs of FENP were observed in 40% (95% CI 34–46%) of the study farms. In addition, the survey clarified the specific clinical signs for different animal species. The presence of disease was associated with the importation of mink, especially from Denmark (OR 9.3, 95% CI 2.6–33.0). The transmission route between Finnish farms was associated with fur animal purchases. Some risk factors such as the farm type were also indicated. As such, FENP was detected more commonly on farms with more than one species of fur animal in comparison to farms with, for example, only foxes (OR 4.6, 95% CI 2.4–8.6), and the incidence was higher on farms with over 750 breeder mink compared to smaller farms (OR 3.8, 95% CI 1.6–9.0). Contact between fur animals and birds and other wildlife increased the risk of FENP on farms. Responses also indicated that blocking the entry of wildlife to the animal premises protected against FENP.

**Conclusions:**

FENP was most likely introduced to Finland by imported mink and spread further within the country via domestically purchased fur animals. Some potential risk factors, such as the type and size of the farm and contact with wildlife, contributed to the spread of FENP. Escape-proof shelter buildings block the entry of wildlife, thus protecting fur animals against FENP.

**Electronic supplementary material:**

The online version of this article (doi:10.1186/s13028-017-0322-z) contains supplementary material, which is available to authorized users.

## Background

In 2007, Finnish fur farmers noticed clinical signs of a novel disease in their animals. Mink (*Neovison vison*) developed necrotic pyoderma on their feet and head, foxes (*Vulpes lagopus*) had severe conjunctivitis that spread aggressively to pyoderma of the eyelids or the facial skin and Finnraccoons (*Nyctereutes procyonoides*, a raccoon dog bred for the fur industry) developed painful abscesses between their toes. The disease continued to spread between and within farms, typical of a contagious infectious disease. It caused severe and even fatal clinical signs that dramatically affected animal welfare and caused considerable financial loss to fur farmers and to the entire fur industry.

Similar lesions in mink were first documented in the USA in 1970s and in Canada in 1996 [1]. Other pelt-producing countries have also reported the disease [2]. In 2010, the Finnish Fur Breeders’ Association (FFBA), Finnish Food Safety Authority (Evira) and the University of Helsinki (UH) initiated a joint project to investigate the macroscopic and histological lesions and to identify the causative agents of the disease. The disease was named fur animal epidemic necrotic pyoderma (FENP) due to the lesions seen in all fur animal species. *Arcanobacterium phocae* in addition to a possible role of a novel *Streptococcus* spp. has been identified as potential causative agents [2]. *A. phocae* has its origin in marine mammals [3], which is compatible with observations by North American farmers who linked similar clinical signs in mink to feeding on seal byproducts [1]. Furthermore, Canadian researchers recently found an association between *A. phocae* and pododermatitis in mink [4]. FENP is potentially a multifactorial disease, where the environment, the host’s immunology and specific infectious agents may be involved. We report here the results of a retrospective epidemiologic survey based on a questionnaire carried out in 2011. The purpose of this survey was to investigate the clinical presentation and farm-level prevalence of FENP in Finland in mink, foxes and Finnraccoon. Our objectives were also to identify potential farm-level risk factors for FENP, to determine how the disease was introduced to Finland and to identify and implement control measures against the disease.

## Methods

### Study design

Data were collected using an epidemiologic questionnaire which was sent to fur animal farms in Finland in either Finnish or Swedish language depending on the language of the farmer. The questionnaire focused on the period 2009–2011 (through the first 6 months of 2011). The questionnaire and a cover letter describing the clinical signs of FENP were sent in the summer 2011 to all fur farms that belonged to FFBA (n = 958). In order to determine the farm-level prevalence of FENP, we aimed for a sample size of 270 farms, assuming 40% disease prevalence at a 5% precision using a 95% confidence interval (CI). This calculation was based on the assumption that farmers held a near-perfect competency in recognizing the clinical signs of FENP [5]. In addition, reminder letters were sent to farmers in August and September 2011. The questionnaire consisted of multiple-choice questions, yes or no questions and questions requiring numerical or written responses. Many of the respondents left portions of the 13-page questionnaire incomplete; eight questionnaires were excluded due to severe incompleteness. To elucidate the response percentage for each variable, the number of missing responses are shown in Additional file 1.

The questionnaire covered the following topics:

1. Clinical signs of FENP (chosen from a list of clinical and visible signs), the year clinical signs were first detected, the species affected, the number of diseased or dead animals due to FENP, color phase of the affected animals, seasonal appearance of the clinical signs, the signs detected and the potential treatment’s effect on FENP-diseased animals.
2. Characteristics of the farm and farmer including the location and the size of the farm according to the number of breeding animals, the housing system, the animal species on the farm, the sex and age of each farmer and the employment of the farmer on the farm as full or part-time.
3. Fur animal imports and purchases.
4. Other diseases on the farm, and prophylactic and therapeutic practices.
5. Fur animal management including sources of food and water, feeding procedures, the drinking water system, bedding materials, manure handling and the storage of succumbed and culled animals before rendering.
6. Biosecurity on the farm including the use of fences, entry of wildlife and birds to animal premises, visitors and traffic, cleaning and disinfection routines and quarantine procedures.

### Definitions of the variables

Data were analyzed by logistic regression. The farms were divided into cases or controls for which risk factors were retrieved from the questionnaire data. A case farm was a farm with FENP in at least one fur animal species during the period 2009 through 2011, while a control farm was a farm with no FENP in any species during the same period. If information about whether a farm was affected by FENP during the study period was missing, FENP presence was inferred using other questionnaire responses, such as procedures used in the treatment of FENP, the number of animals that had died or recovered from FENP and medication used to treat FENP.

Farms were divided into four types: (1) mink farms (solely mink), (2) fox farms (solely foxes), (3) Finnraccoon farms (solely Finnraccoons) and (4) mixed farms (at least two species). In cases where an insufficient response to the question concerning farm type was provided, farm type was inferred from the information provided regarding the number of mink, foxes and Finnraccoons on the farm, medication and vaccines administered to different species and purchases or shelter buildings specified for species.

We categorized farms as small or large according to the number of breeder animals: mink, >750 vs ≤750; fox, >320 vs ≤320; and Finnraccoon, >125 vs ≤125. In farms with more than one species, size was based on the most numerous species. For example, a farm with 600 mink and 200 Finnraccoons was considered a small farm because there were more mink than Finnraccoons, and fewer than 750 mink.

In some cases, it was necessary to study variables for all farms that had mink, foxes or Finnraccoons regardless of whether there were one or several species on the farm. In these cases, the farms were referred to as “farms with mink”, “farms with foxes” and “farms with Finnraccoons” to distinguish them from exclusive mink, fox and Finnraccoon farms.

We collected information at annual level, although for some events it was sufficient to know if an event had occurred at all during any of the years in question. Therefore, for variables regarding diseases other than FENP, medication given, vaccinations administered and purchases and the importation of animals, we created new combined variables that summed annual data appropriately. If necessary, both annual and combined variables were analyzed. All variables are listed in Additional file 1.

### Statistical analysis

The included farms and farmers (i.e., respondents) were compared with all Finnish fur farms and farmers to determine the representativeness of the study population. Information on all Finnish fur farms was obtained via a questionnaire distributed by FFBA in 2010. The distributions for different species, the geographical distribution of the farms and the main characteristics of the farmers were compared statistically.

The frequencies of all variables in the study were counted in groups of cases and controls. Crude odds ratios (OR, with only one independent variable in the model at a time) and their 95% confidence intervals (95% CIs) were calculated for all variables. A multivariable logistic regression analysis was performed on four groups based on the animal species composition of the farm: (1) all farms, (2) mixed farms only, (3) farms with mink (also including mixed farms with mink) and (4) farms with foxes (also including mixed farms with foxes). For each group, the variables with a significant crude OR at 95% confidence level were included in the model. We tested the effect of missing values by running models that also included missing values for variables recoded as “no”.

Multicollinearity was tested using the phi coefficient for binary variables [6] and by considering wideness of CIs in occasion of more than two category variables. Interactions up to the second order were tested between all variables. The Pearson’s goodness-of-fit statistic (Pearson GOF) and the McFadden’s and Cox and Snell R^2^ statistics [7, 8] were used to identify the most parsimonious model.

All statistical analyses were performed using SAS version 9.3 (SAS Institute, Cary, NC, USA). The Proc Freq statement with the Chisq and Fisher options was used to test differences and associations among categorical data. The Proc Npar1way statement with the Wilcoxon option (producing the Kruskal–Wallis test) was used for data with more than two categories. The Proc Logistic statement was used for the logistic regression analysis to define the most important factors in the transmission of FENP to the farm and its further spread on a farm.

## Results

### Description of the study farms

**Fig. 1:**
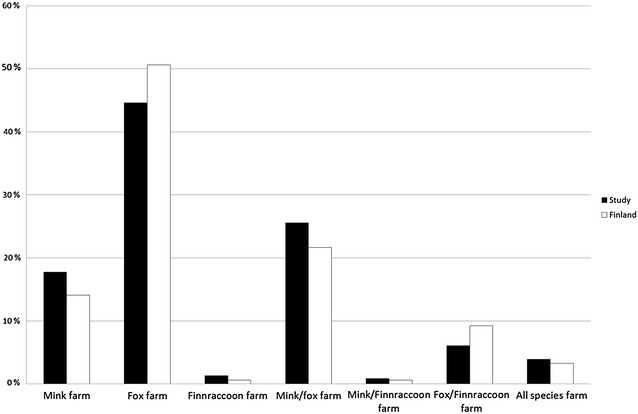
Characteristics of the fur farms included in the study and all Finnish fur farms (2010). Farms in the study compared with all farms in Finland according to the fur animal species farmed. The information for Finnish fur farms was obtained from the Finnish Fur Breeders’ Association (FFBA)

**Fig. 2:**
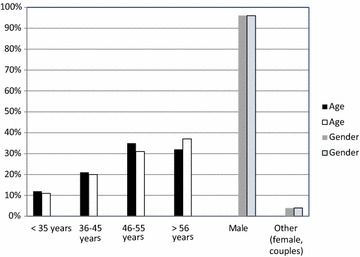
Characteristics of fur farmers included in the study and all Finnish fur farmers (2010). The age and gender of the fur farmers in the study compared to fur farmers in Finland. The information for Finnish fur farmers was obtained from the Finnish Fur Breeders’ Association (FFBA)

Questionnaires were returned by 239 farmers (25%). A comparison of the farms included in the study and all Finnish fur farms showed that study farms and farmers well represented fur farms and farmers in Finland in general based on the farm type (Chi square, P = 0.71), age group (Kruskal–Wallis, P = 0.29) and gender (Chi square, P = 0.96) of the farmers (Figs. 1, 2).

In Finland, 97% of fur farms are located in the western part of the country [9], which was similar to the geographical distribution of the respondents’ farms (94%). Most farms in this study had traditional shelter buildings, seven had both shelter buildings and halls and only two had halls alone. Standard cages were used for animal housing. Due to new legislation on fox cage sizes (introduced 1 January 2011), 81% of fox farms had recently changed their cages. The distance between individual farms varied. We found that about half were more than 500 meters apart (Additional file 1).

Most farmers were men, while only a few were women or farmed by a couple. About half of the farmers were under 50 years old, and the age ranged from 22 to 71 years (Fig. 2). On 80% of farms, fur farming was reported as the main occupation (data not shown).

### Occurrence of FENP in the study farms

**Fig. 3:**
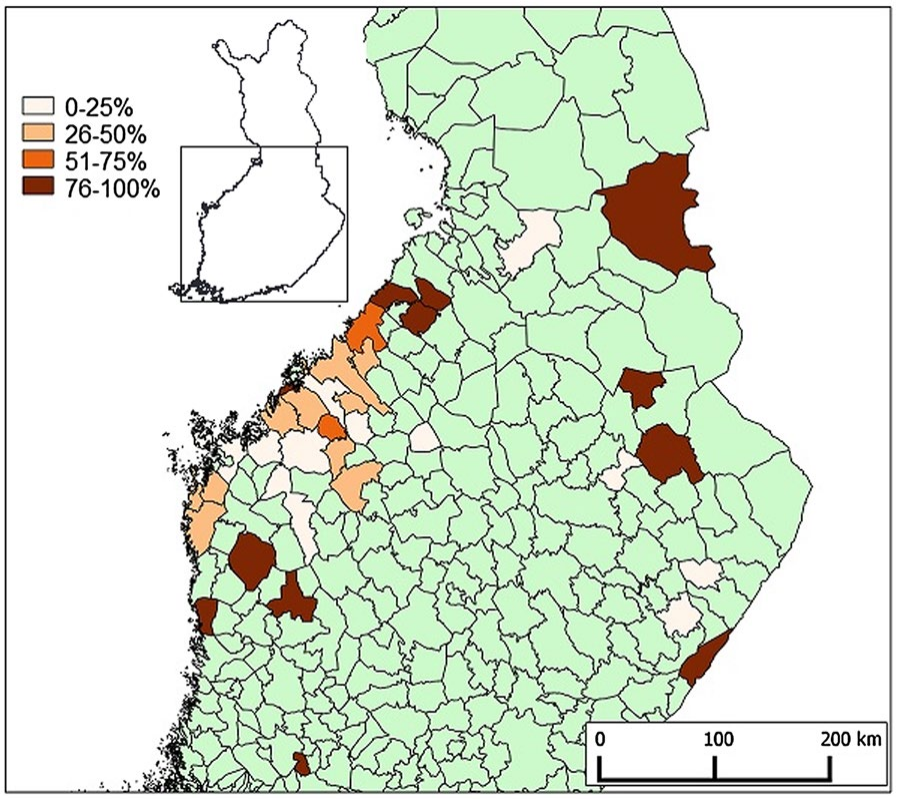
Fur animal epidemic necrotic pyoderma (FENP) on participating farms. The geographic distribution of the farms and percentage of farms reporting FENP during the period from 2009 through 2011. Areas in *green*: no participants

**Fig. 4:**
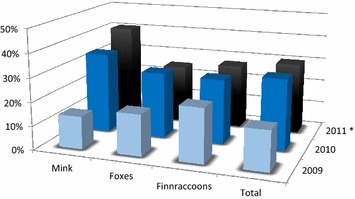
Occurrence of fur animal epidemic necrotic pyoderma (FENP) on Finnish fur farms. Occurrence of FENP in mink, fox, Finnraccoon and all study farms during the period from 2009 through 2011. *Asterisks* first 6 months of 2011

The survey showed that FENP had spread to all areas where fur farming is practiced in Finland during the period from 2009 to 2011 (Fig. 3). Clinical signs of FENP were detected on all fur animal species. FENP was reported by 92 (40%; 95% CI 34–46%) of the responding farms, including 16 (39%; 95% CI 26–54%) mink farms, 25 (24%; 95% CI 17–33%) fox farms and 51 (61%; 95% CI 50–70%) mixed farms. The number of affected farms increased during each study year (Fig. 4).

New animals had been bought either from domestic markets or imported from other countries by 89% of all study farms. More specifically, 93% of the farms that reported clinical signs of FENP and 88% of the farms without clinical signs of FENP bought new animals. Animals were imported by 24% of all study farms, by 35% of farms reporting clinical signs of FENP and by 18% of farms without clinical signs of FENP (Additional file 1). Farms with clinical signs of FENP imported more mink from Denmark than farms without clinical signs during all study years (Table 1).

**Table 1:** Fur animal imports by the study farms

Country of origin^a^		Denmark					Poland					Norway				
	FENP	Yes (%)		No (%)		P^b^	Yes (%)		No (%)		P^b^	Yes (%)		No (%)		P^b^
2009	+	9 (24)		29 (76)		0.001	0 (0)		38 (100)		1.000	1 (3)		37 (97)		1.000
	–	7 (5)		141 (95)			1 (1)		147 (99)			7 (5)		141 (95)		
2010	+	12 (18)		56 (82)		0.011	5 (7)		63 (93)		0.536	3 (4)		65 (96)		0.396
	–	8 (6)		130 (94)			7 (5)		131 (95)			11 (8)		127 (92)		
2011	+	5 (7)		62 (93)		0.044	9 (13)		58 (87)		0.001	3 (4)		64 (96)		0.394
	–	2 (2)		130 (98)			2 (2)		130 (98)			12 (9)		120 (91)		

Fur animal imports from Denmark, Poland and Norway during the period from 2009 through 2011 for FENP-positive (+) and FENP-negative (−) farms

^a^In 2010 and 2011 combined imports of the year in question and the previous year because of the unknown incubation time of FENP

^b^Fisher’s exact test P value

In 2009, farms purchasing from domestic sources alone had significantly more FENP than farms without any purchases. However, when the entire period from 2009 to 2011 was included in the analysis, domestic purchases did not significantly increase risk (OR 3.7, 95% CI 0.8–17.1; Table 2).

**Table 2:** Domestic fur animal purchases by study farms

	FENP	Domestic purchases^a^				
		Yes (%)		No (%)		P^b^
2009	+	26 (100)		0 (0)		0.002
	−	109 (71)		45 (29)		
2010	+	44 (94)		3 (6)		0.052
	−	103 (82)		23 (18)		
2011	+	47 (94)		3 (6)		0.091
	−	110 (85)		20 (15)		

Domestic fur animal purchases for the period from 2009 through 2011 among FENP-positive (+) and FENP-negative farms (−)

^a^In 2010 and 2011, we combined purchases for the year in question and the previous year because of the unknown incubation period for FENP

^b^Chi square test, P value

Farms with clinical signs of FENP sold animals to other farms more than those farms without clinical signs of FENP (FENP + 37% vs FENP −28%); however, the difference was not statistically significant (Chi square, P = 0.14).

Only 25% of all farms that had imported animals used some form of quarantine on their farm. Animals purchased from Finnish farms were kept in quarantine by 14% of farms. In addition, fences were built to enclose the animal premises to avoid fur animals escaping and to keep wildlife from entering the farm on 54% of mink farms, 60% of farms with foxes and 86% of farms with Finnraccoons. According to respondents, wildlife and birds had access to many farms. Both birds and other wildlife were seen inside the farm significantly more often on fenced farms with mink than on unfenced farms with mink (Fisher’s exact test, P < 0.0001 and P = 0.001, respectively), while birds were detected inside the farm significantly more often on fenced farms with foxes than on the unfenced farms with foxes (Chi square test, P < 0.0001; Additional file 1).

In addition to FENP, farmers also reported other diseases on their farms. Among these diseases, pre-weaning diarrhea (“sticky kits”) (42%), plasmacytosis (32%) and urolithiasis (25%) were most frequently reported on mink farms. Among foxes, the most common diseases consisted of conjunctivitis (55%), fertility disorders (abortion 32%, endometritis 23%) and cystitis (51%). Diarrhea, which is one of the most common diseases, was not included in the options listed on the questionnaire for foxes. In Finnraccoons, the most common diseases included parvovirus enteritis (11%) and abortion (11%). The most commonly used medical treatments on the farms responding were penicillin as an injectable antibiotic and tetracycline, lincomycin and ivermectin (foxes and Finnraccoons) mixed in with feed (data not shown).

Cleaning routines varied between farms. As such, 30% of farms had their own defined schedule of regularly washing cages, 19% washed their cages only after a disease outbreak and 21% of farms did not wash the cages at all. Disinfection was performed regularly (based on the farm’s own schedule) on 7% of farms, after a disease outbreak on 30 and 41% did not disinfect the cages at all. Over half of the farms used drinking nipples (80% of mink farms, 44% of fox farms and 68% of mixed farms), while the rest used cups or both nipples and cups. Only mink farms had beddings in nests relying on several different materials (e.g., straw, saw dust, hay, shavings and turf; Additional file 1).

On affected farms, farmers reported signs of pyoderma in the head and on the feet of affected mink. Discharge from the eyes and pyoderma in the head was reported as affecting foxes. Affected Finnraccoons experienced lesions on the paws (Table 3).

**Table 3:** Clinical signs of fur animal epidemic necrotic pyoderma (FENP) in mink, foxes and Finnraccoons

Clinical signs	Mink (n = 32)		Fox (n = 47)		Finnraccoon (n = 6)	
	n	(%; 95% CI)	n	(%; 95% CI)	n	(%; 95% CI)
Periocular	1	(3; 0–16)	33	(70; 56–81)	0	(0; 0–39)
Head	14	(44; 28–61)	26	(55; 41–69)	0	(0; 0–39)
Paw	28	(88; 72–95)	2	(4; 0–14)	6	(100; 61–100)
Other parts	6	(19; 9–35)	3	(6; 0–17)	1	(17; 3–56)

Farmers reported having performed the following procedures on FENP diseased animals: medication only, culling all diseased animals without any medication only or both medicating and culling. To treat FENP, farmers primarily used penicillin as an injectable antibiotic in animals with a diminished appetite and oral administration of tetracycline and lincomycin mixed in with the feed in other animals. In addition, when a parasitic skin disease was assumed before the diagnosis of FENP was established, medication with ivermectin was used to treat foxes and Finnraccoons. Farmers reported that animals benefitted from medication with antibiotics, particularly penicillin (data not shown). However, only a few farms (n = 31) having animals with clinical signs of FENP used medication instead of culling.

### Risk factors for FENP on the study farms

A clear connection between the incidence of FENP and importing mink from Denmark and Poland emerged compared to farms that did not import from these countries. By contrast, imports from Norway and the USA were not associated with increased risk of FENP (Table 4; Additional file 2).

**Table 4:** The crude odds ratios of relevant risk factors for fur animal epidemic necrotic pyoderma (FENP)

Risk factor	Case farms (n = 92)		Control farms (n = 134)		OR (95% CI)	
	Exposed (n)	Non-exposed (n)	Exposed (n)	Non-exposed (n)		
All farms						
Farm type						
Mixed farm vs mink farm	51	33	16	24	2.3 (1.1–5.0)	
Mixed farm vs fox farm	51	33	25	74	4.6 (2.4–8.6)	
Purchases						
Domestic purchases	86	2	118	15	3.7 (0.8–17.1)	
All imports combined	32	56	24	96	2.3 (1.2–4.3)	
Imports from Denmark	17	71	3	117	9.3 (2.6–33.0)	
Imports from Poland	10	78	2	118	7.6 (1.6–35.5)	
Drinking system						
Cup	22	68	58	72	0.4 (0.2–0.7)	
Nipple	67	23	66	64	2.8 (1.6–5.1)	
Farms with mink (including mixed farms)						
Fence around the mink premises	38	19	21	28	2.7 (1.2–5.9)	
Access by birds to shelter buildings	25	34	10	38	2.8 (1.2–6.7)	
Access by wild animals to shelter buildings	13	42	2	43	6.7 (1.4–31.3)	
Size of the farm: >750 vs ≤750 breeder mink	39	13	19	24	3.8 (1.6–9.0)	
Hay as bedding material	9	48	18	31	0.3 (0.1–0.8)	
Pre-weaning diarrhea	31	30	15	34	2.3 (1.1–5.2)	
Plasmacytosis	13	48	23	26	0.3 (0.1–0.7)	
Farms with foxes (including mixed farms)						
Access by wild animals to shelter buildings	22	44	18	75	2.1 (1.0–4.3)	
Size of the farm: >320 vs ≤320 breeder foxes	44	20	44	54	2.7 (1.4–5.2)	
Mixed farms						
Size of the farm large vs small (based on the most numerous species)	26	18	13	19	3.0 (1.2–7.9)	
Fence around mink premises	33	9	12	13	4.0 (1.4–11.7)	

Number (n) and crude odds ratios (OR, with only one factor in the logistic regression model at a time) for the most prominent risk factors for FENP

Larger sized farms (according to the number of breeding animals) had a higher risk of FENP than smaller sized farms across all farm types. However, this risk was not significant on farms with solely mink or solely foxes (Additional file 2). Nearly half of the farms with more than 750 breeder mink had imported animals, whereas the percentage on smaller mink farms having imported animals was 26%. However, the difference in imported animals between different sized farms was not significant (Chi square test, P = 0.079).

Mixed farms had a higher risk for FENP than farms with only mink or only foxes (Table 4). Mixed farms with one FENP affected species also had a higher risk for FENP in other fur animal species on that farm. For instance, in 2010, when mink on a farm were FENP affected, foxes on the same farm had a 22-fold higher risk of developing FENP-positive compared to farms with no diseased mink (data not shown). The occurrence of FENP on farms with mink was associated with wildlife contact. For instance, a significant risk on farms with mink was associated with contact to wild animals and birds compared to farms without such contact. Furthermore, farms using fences around the shelter buildings had a significantly higher risk of FENP than farms with no fences.

Pre-weaning diarrhea [10] exhibited a significant positive association and plasmacytosis in mink exhibited a negative association with development of FENP, whereas feeding with on-farm formulated or commercial feed, feeding procedures or water sources were not associated with FENP. Mink farms that used hay as bedding material had a significantly lower risk for FENP than farms that used other bedding materials. Cleaning and disinfection routines and vaccinations on farms had no effect on the occurrence of FENP (Table 4). Clinical signs of FENP were detected in all color phases, but were least common in the brown color phase in mink. Within foxes, blue foxes were more frequently affected by FENP based on the frequency data from the study (data not shown).

The results of the multivariable logistic regression analyses are presented in Table 5. The best model for all farms (model 1) included the variables “farm type”, “imports from Denmark” and “imports from Poland”. The best model for mixed farms (model 2) included the variables “bird access to mink farm” and “nipple drinking system”. The best model for all mink farms (model 3) included the variables “imports to the farm during 2009–2011”, “size of the farm” and “wildlife access to mink farm”. Finally, the best model for all farms with foxes (model 4) included the variables “farm type”, “wildlife access to mink farm” and “nipple drinking system”.

**Table 5:** Multivariable logistic regression analyses of significant risk factors for fur animal epidemic necrotic pyoderma (FENP)

Model	Cases (n)/	Risk factors	OR (95% CI)	Goodness-of-fit statistics		
	Controls (n)			Test	Value	P
Model 1	88/118	Farm type				
		Mink farm vs fox farm	1.3 (0.5–3.1)	McFadden’s R^2^	0.147	
		Mixed farm vs fox farm	3.8 (1.9–7.6)	Cox-Snell R^2^	0.182	
		Imported from Denmark	6.0 (1.6–22.8)	Pearson	0.721	0.608
		Imported from Poland	7.2 (1.4–37.3)			
Model 2	42/23	Access by birds	4.6 (1.2–16.8)	McFadden’s R^2^	0.188	
		Nipple	8.4 (2.0–35.0)	Cox-Snell R^2^	0.217	
				Pearson	0.411	0.675
Model 3	42/34	Imports	5.3 (1.6–18.0)	McFadden’s R^2^	0.241	
		Access by wildlife	13.6 (1.5–121.0)	Cox-Snell R^2^	0.282	
		Size of the farm > 750 vs ≤ 750 mink	3.1 (1.0–9.0)	Pearson	0.561	0.847
Model 4	65/90	Mink farm vs fox farm	4.5 (2.1–9.4)	McFadden’s R^2^	0.179	
		Access of wildlife	2.3 (1.0–5.4)	Cox-Snell R^2^	0.216	
		Nipple	3.3 (1.6–7.0)	Pearson	0.658	0.621

*Model 1* all farms, *Model 2* mixed farms *Model 3* farms with mink *Model 4* farms with foxes

Number (n) of case farms and control farms in the model and odds ratios (OR) of the variables included in the model. In all of the goodness-of-fit tests, a test value of 1 indicates a particular well-fitting model; a Pearson’s value <0.05 indicates which model should be rejected. The variable “farm type” has three categories: mink, fox and mixed farms where fox farms serve as the reference group

All of the variables included in the models were significant risk factors for FENP, except for the variable “farm type” when comparing mink farms to fox farms in model 1. We detected no interaction and only a slight multicollinearity between the variables included in the models. Instead, severe multicollinearity was found between the use of fences around animal shelters and wildlife and bird access to animal shelters, indicating a strong association between these variables.

## Discussion

In 2007, Finnish fur animal farms experienced a novel disease designated as FENP. This study revealed that FENP is a detrimental disease in Finnish fur animals and appeared to have spread within and between farms over the study period. The disease also caused severe, sometimes lethal, disease, thereby clearly adversely impacting animal welfare and causing considerable financial loss to farmers.

The clinical signs reported by farmers included pyoderma on the head and feet of mink, conjunctivitis which spread to an inflammation of the eyelids and facial skin areas of foxes and the development of abscesses on the paws of Finnraccoons. These clinical signs are consistent with previous observations in a Finnish study that described the clinical outcome and pathological findings of FENP [2].

Farmers suspected that FENP originally arrived in Finland via imported fur animals. We found an association between the occurrence of FENP and mink importation, particularly from Denmark. Farmers suspected that the further spread of disease in Finland was connected to animal purchases between Finnish fur farms. This putative mechanism agrees with our finding that farms affected by FENP purchased more from domestic sources, particularly in 2009. Our study indicated that quarantine procedures were not a common practice on Finnish fur farms during the outbreak. Thus, in order to avoid the spread of infectious diseases, sufficient quarantine procedures are also crucial on fur farms.

In addition, our study showed that wildlife and birds may act as carriers of FENP, thus spreading the infection on as well as between farms. Unexpectedly, our results showed that FENP was more often detected on farms enclosed by a fence. However, according to the Finnish certification system, farms without a fence are required to construct shelter buildings that entirely prevent fur animals from escaping the premises (escape-proof shelter buildings). Escape-proof shelter buildings much more effectively hinder wildlife access to animal premises and better block contact between wildlife and fur animals than fences. This indicates that high-level biosecurity procedures including the control of wildlife access to farms could limit the spread of FENP.

The FENP risk varied between different types of farms. Farms with a higher number of breeder mink also exhibited a higher risk of FENP. In addition, larger mink farms imported more animals than smaller farms. However, both the size of mink farms and the importation of animals independently affected the risk for FENP. Mixed farms with more than one fur animal species had a higher risk of FENP than farms with only one species. We found that if one species on a mixed farm was FENP-positive, then other species experienced a higher risk for FENP infection. It may be that FENP susceptibility varies across species and some species may spread disease without showing obvious clinical signs. Furthermore, in mink the different color phases seemed to carry different risks for acquiring FENP. In general, FENP was detected in all color phases, but the brown phase, known as the most vigorous [11], appeared to accompany more resistance to FENP on the farms.

FENP was associated with the occurrence of other diseases. For example, pre-weaning diarrhea in mink occurred more on farms that also had FENP. Pre-weaning diarrhea is a multi-causal disease where viral, bacterial, environmental and dietary factors are all involved [10]. Mink that survive pre-weaning diarrhea may hypothetically be weaker and more prone to other diseases due to a compromised immunity. Alternatively, pathogens causing FENP could already be present at birth, even when no typical lesions are present, thus predisposing minks to pre-weaning diarrhea. It is also possible that similar environmental factors, such as hygiene and management, serve as predisposing factors in the occurrence of both diseases. Surprisingly, farms with plasmacytosis carried a lower risk of FENP. Plasmacytosis (Aleutian disease) is a parvoviral mink disease that attenuates the immune system, thereby predisposing animals to other diseases [12, 13]. There is no effective vaccination against plasmacytosis. However, serological screening and control systems conducted on Finnish mink farms [14] divide farms into categories based on the plasmacytosis seroprevalence. It may be that plasmacytosis-positive farms do not check their animals as thoroughly as plasmacytosis-negative farms, whereby they missed some cases of FENP believing that death or clinical signs resulted from plasmacytosis.

Feed might represent one potential source of acquiring FENP. For instance, North American farmers and researchers [1] linked the onset of similar clinical signs to feeding mink with seal byproducts. However, in the current study we found no association between feed sources or feeding systems and the detection of clinical signs of FENP. The original source of the epidemic has probably been seal meat in the mink feed, but due a species shift of the causative agent from marine mammals to mink, FENP is currently transmitted between fur animals [2]. Very little variation occurs in the raw materials utilized in all feed kitchens and no seal byproducts are used in Finland. Clinical signs of FENP were also detected more often on farms that used drinking nipples instead of cups. In general, a nipple water dispensing system is considered more hygienic than a cup system. However, the cups are cleaned regularly whereas the nipples are cleaned less often. Contact between oral mucous membranes and nipple structures might thus act as a predisposing factor for FENP. Furthermore, occasionally cages have sharp wires that can cause wounds, and any sharp protruding structures, such as wires, near the drinking nipple could cause skin trauma, particularly to the head or the feet when an animal is drinking. Experimental infection of mink with *A. phocae* indicated that a skin trauma is needed to transmit the infection [15]. Thus, the role of feed as a possible source of infection and a nipple drinking system as a predisposing factor require further investigation.

We also found that various types of bedding materials were used on mink farms. Hay seemed to protect against FENP. This result is, however, controversial since hay can also cause problems whereby the thick and sharp coarse stalks can traumatize the mucosa in the mouth and cause abscesses, especially when mink are in the sapphire color phase [16]. Differences in the quality of the hay used on the farm exist and softer, less coarsely textured hay may not possess these negative side effects. We did not, however, specifically ask about the hay quality. In addition, hay may also provide solid insulation in the nests and protect against the cold, another external stressor affecting mink.

## Limitations

This study carries certain limitations. We cannot completely eliminate the possibility of a nonresponse error [17], since farms that had FENP may have been more motivated to participate in the survey. Alternatively, these same farms might have avoided participating due to the fear of being identified, despite the anonymity of their responses. Responding to this comprehensive questionnaire was time-consuming, thus potentially lowering the response rate and leading to incomplete responses. This may have caused an information bias due to some of the inferences we had to make as well as a measurement error in our results [17]. Despite the low response rate, however, the survey responses adequately represented Finnish fur farms, and included diseased and non-diseased farms (Figs. 1, 4).

Furthermore, it is possible that the descriptions of the clinical signs of FENP provided prompted farmers to report more cases than they actually had. It is, however, also possible that some of the reported cases of FENP in this study were misdiagnosed by farmers since other skin lesions such as biting wounds occur in mink [18]. In foxes, entrophia or ectopic cilia [19] may cause eye inflammations resembling FENP, while the clinical signs in these diseases are much milder. However, we assumed that the farmers were well informed of and competent in recognizing the clinical signs of FENP.

## Conclusions

Our study provides further evidence that FENP is a detrimental disease to Finnish fur farms. We found that FENP was likely to have been introduced to Finland via imported mink, particularly those imported from Denmark, and then spread to other farms via domestic purchases and the transfer of infected animals. Other possible causes of the spread of disease between animals and to other farms included fur animal contact with wildlife and birds. FENP occurred more on larger farms and on mixed farms, and one diseased species increased the risk of cross infection to other fur animal species on the same farm. These results provide areas of focus for control measures against FENP.

## Additional files

**Additional file 1.** Frequencies of all variables included in the study in groups of all farms/mink farms (solely mink)/fox farms (solely foxes)/Finnraccoon farms (solely Finnraccoon)/mixed farms, all farms/mink farms (solely mink)/fox farms (solely foxes)/Finnraccoon farms (solely Finnraccoon)/mixed farms with FENP and all farms/mink farms (solely mink)/fox farms (solely foxes)/Finnraccoon farms (solely Finnraccoon)/mixed farms with no FENP.

**Additional file 2.** Crude odds ratios (OR) with 95% confidence intervals (CI) of all variables included in the epidemiologic study of FENP in Finland: All farms, mixed farms, mink farms, fox farms and adjusted by the farm type.
